# Dental Education

**Published:** 1851-10

**Authors:** Charles W. Ballard

**Affiliations:** 872 Broadway, New York.


### ARTICLE VI.

*Dental Education.* By CHARLES W. BALLARD, M. D., D. D. S., 872 Broadway, New York.

IT would, perhaps, be well, before suggesting *improvements* upon the present system of instruction in dental science, to make known to the profession generally, the advancement which has been made upon the methods of education, adopted in former years, together, with the various causes which, acting separately or combined, have rendered such improvements necessary. The proper accomplishment of this object, seems to require, at the least, a brief history of dental education, as it has existed in this country, together with some remarks relative to the effects, which its different phases may have had, upon the *public*, as well as upon the profession.

Up to the year 1810, there were but few dentists practicing in this country, and these few had principally obtained the knowledge they possessed of the art, from practitioners in Europe. They were, so far as we are able to judge, all of them, men of some ability, and as well acquainted with the duties devolving upon them, as the existing state of dental science permitted. No works upon operative or mechanical dentistry had been written in this country, and but few in France; in England two or three valuable works relative to the teeth and their treatment had just appeared. It was, however, exceedingly difficult to procure any of these works here, and it was many years before any work of importance to the student of dental surgery was published in the United States. In the mean time, the demand for dentists increased. The few who may be said to have given the profession its first start in this country, were generally doing a good practice; their time was well employed, and their services appreciated. The majority of them, we believe, received students in their offices and taught them the practical part of the art, which consisted, at that time, of carving ivory blocks, extracting and occasionally filling teeth.

Owing to the absence of American dental literature, and the scarcity and expense of that published in Europe, it became a matter of necessity that those who, at this time, studied in the United States, should be in many of the more scientific points relating to the profession, most sadly deficient. This was a difficulty in the way of dental education, which could only be overcome by rendering dental works more plenty, a thing more easily talked about than accomplished, as is evident from the fact that though the evil was known and felt, it was some years before the remedy was commenced, and many more had elapsed ere it could be said to be fairly under weigh. During this time the number of dentists practicing in different parts of the country was fast increasing; by the year 1825, they numbered a little over two hundred—of these some had obtained thei rknowledge of the profession from those first spoken of; but the majority had commenced practicing without any claim upon the public beyond that of having purchased a few secrets from such as possessed them, and were willing to sell, depending upon these secrets, and their own ingenuity and boldness, to help them into practice and out of difficulties.

As late as the year 1830, the means of obtaining information upon such subjects as related to dental surgery, were extremely limited. It is true books were beginning to be published, but they were mostly small popular works, and generally added more to the reputation of the authors than to the available literature of the profession. They were, however, productive of much good, by drawing the attention of the public to the importance of preserving the teeth.

In all of our large cities were to be found some few men, who having possessed themselves of what might be called the theory of dental science, had put it in practice, and by much energy, perseverance and skill, were enabled to add greatly to the information which had been imparted to them by their instructors. These men became teachers in their turn, but like those who had trodden the ground before them, their time and skill were needed and demanded by their patients, and the number of students to whom they could give *proper attention*, was by far too small to supply the demand for dentists, and a host of pretenders rushed in to supply the deficiency. Dental surgery may with truth be said to have been at this time at its lowest ebb. Fortunately for us, it proved to have been only that dark hour which precedes the light of day.

The various methods in vogue, at this time, of obtaining a knowledge of the profession, or of founding claims upon the public as dental surgeons, may, together with the dentists thus constituted, be set down in three distinct classes.

Class first—consisted of those whose ignorance was their only excuse for the injuries they inflicted upon their patients, and ultimately their profession, as also of those, who having purchased or traded for a secret or two, depended upon bold faced and unblushing impudence for success. Very many availed themselves of their *natural advantages* in this respect, to obtain from the public a support, and in some instances as much more as would suffice to give them in the later years of their lives, a *separate maintenance.* Such men could only stand high in *their own* estimation, by dragging the profession down to their own level, and then trampling it under their feet, proclaim to the world that they were "neither uncommon or unclean," "they were not as other men." "They stood in high places and made long prayers for a pretence." These were the Pharisees of the profession. They could be best judged by their works, provided such judgment was as hasty as a coroner's inquest, as their works were exceedingly transient, save and excepting their work of demolition. Dentists to this day, suffer to some extent, from the odium brought upon their calling by the acts of these men. Those of the public who had suffered from their ignorance, resented the injury by closing their doors to the whole profession. A dentist soon came to be despised, the name was a reproach, and those dentists who held a favorable position in the opinion of the public, owed it more to their character and reputation as individuals than to their abilities as dental surgeons.

Dentists, of the class I have been describing, knew little and cared less, about the duties devolving upon them, and yet they were always ready to receive pupils and instruct them in the *secrets* and *mysteries* of dental science, provided they were were well paid for it. The fees exacted in these cases, varied from five dollars to one thousand, and the amount of information imparted varied quite as much, though it bore no relative proportion to the sum paid. The length of time occupied by these initiatory proceedings, depended very much upon the ability of the student, and the ignorance of the teacher. It was generally conceded by these dentists, that the shorter the time wasted in this manner, the better for all parties, and the teachers were *always willing to communicate as little as possible.*

The second class, may be considered as consisting of those dentists, who having obtained as great a knowledge of the principles and practice of dental surgery, as their time, means or opportunities would allow, came at once to the conclusion, that so long as they did the best they knew how for their patients, and comported themselves in other respects as became good citizens, that they had done their whole duty, and neither the public or the profession had any further demands upon them. With this class of dentists, may be included those who commenced practice with little or no education, and were compelled, in order to compete with those around them, to add, by every means in their power, to the knowledge and experience that their practice was daily giving them. Many of these men, eventually became, to a certain extent, good practitioners, but of the best of them, it would be difficult to say whether the good or the evil which they had done in their day preponderated.

Dentists of the second class, were much better acquainted with their professional duties than those first described ; and very many of them excelled in that branch of the practice, known as mechanical dentistry ; and in justice to them it must be borne in mind, that at the time of which we are writing, mechanical dentistry was considered, by a majority of the profession, to be, by far, the most important part of dental practice.

Those who gained their knowledge of the practice of dentistry, in the offices or laboratories of dentists of this class, necessarily partook more or less of the characteristics of their teachers ; certainly they could not then and there, take rank above them. To be sure, they stood higher in the scale than those who might be called the successors of the first class, nevertheless, they had much to attain and many obstacles to overcome before they could render themselves really useful to the public. It was the custom for the beginner, after he had passed through the usual course of instruction, and before he had commenced offering his services to the public, to spend some length of time in the office of his teacher, attending to the plainer duties of the mechanical department, and in some cases, doing the whole of the mechanical work. In many instances, this extra work was done for the purpose of liquidating the debt entered into at the start—(dentists being often made on credit)—others did it for the sake of embracing every opportunity of improving themselves, and others, again, fearing to encounter alone the troubles and difficulties always experienced by young men just entering the practice of a profession, did it for a support, while waiting, like Mr. Micawber, "for something favorable to turn up."

This course of procedure, undoubtedly, added much to their skill in that department of practice, and eventually enabled them to come before the public much better qualified to act as dentists than those who had adopted the more headlong course.

The relative proportion which these various classes of dentists bore to each other, may safely be set down to about one-half of the former to three-eighths of the second, and one-eighth of the third classes.

Dentists of the third class, although numerically less than either of the other classes, had, as a result of their course of practice and deportment generally, acquired far more reputation and influence. They had commonly obtained their knowledge of the profession by studying in the offices of such men as were in good practice and possessed a fair reputation in the localities where they practiced. Had these beginners stopped here, and settled down, after the manner of their teachers, no further description of them would have been rendered necessary. Fortunately for dental science, this was not the case. These few men seemed, from the outset, to have been impressed with the belief, that the resources of the science were by no means developed—that dental surgery held a position far beneath that to which it was entitled—that the odium which had been cast upon the profession was a natural result of the ignorance and unfounded pretension of a majority of its members, as well as a want of discrimination on the part of the public generally, between the perfect or imperfect performance of the various duties devolving upon the surgeon dentist; and, lastly, as all these evils could and should be remedied, it was their duty to devote a portion of their time and energies to the good work.

Unquestionably, the first step they could take, was to improve themselves; to become thoroughly acquainted with dental surgery as it was then practiced in this country and in Europe; to carefully sift the good from the bad; to give truths a more prominent position and to expunge all errors. This required years to accomplish. That which had been acquired by study had to be tested by experience, ere it could receive its proper value as a practical fact. The next thing to be done, was to make the profession acquainted with the result of their labors. Consequently, dental literature, of an improved character, began to make its appearance. This change at once advanced the profession. Many of the dealers in secrets lost immediately a most important part of their traffic; they soon had very few secrets to sell, and a still smaller number of purchasers. Consequently, they were obliged either to give up wholly the practice of dentistry, or to confine their attention to it, to a degree such as nothing short of a prospect of losing their means of support could ever have compelled them. This was a great gain of itself, but much more was to be done. The few had presented to the profession their many facts, but a vast amount of valuable information was still treasured up by those who, from their ingenuity or experience, had become possessed of it. Every practitioner of standing had some theory or fact to add to the general fund ; not enough, perhaps, to fill a book, but still, far too much to be lost ; each one was anxious to give, and all were anxious to receive. Hitherto, the members of the profession had held aloof from each other, regarding those who proposed free interchange of thoughts and experience as spies, disposed to impart as little as possible, for the sake of gaining all the knowledge they could.

A reaction in feeling was now taking place, and to encourage it and facilitate the intercourse between members of the profession residing in different states, the American Journal of Dental Science was established. To this was also added the Library, which was nothing more or less than the periodical republishing of the principal works upon dental surgery which had appeared in Europe up to that time. The Journal and Library united, formed a most valuable addition to the reading matter now placed before the profession, and there were, comparatively, few members of it who did not avail themselves of the information contained in its pages. The benefits resulting from this were, undoubtedly, very great, and must have been most gratifying to those who had been endeavoring for years to advance the interests and standing of the profession.

By this time, a complete revolution had taken place in relation to the comparative importance of the two branches of dental practice. Mechanical dentistry, which had hitherto been considered as the most important branch, now gave place to the operative department; it was now considered far more important, to arrest disease in its onward progress, than merely to hide its ravages. Operations upon the mouth and teeth, became now the most important part of dental practice; but to become a skillful and scientific operator, required an expert-

6*

ness in manipulation, that could only be acquired by actual practice upon the living organs, and that too, when guided by a quick eye, a sound judgment, and a thorough knowledge of the importance of the duties to be performed.

At this time, the best means by which a dental education could be had, were not much superior to those of previous years. It is true, books were to be had, and of the best kind. They were doubtless of great importance. They contained a large amount of information that was to a beginner invaluable. They did much towards providing him with a matured judgment, and a thorough knowledge of the organs upon which he was to operate, but they could not give him manual dexterity and skill. The question arose, as to where and how this one thing needful could be obtained. The mechanical branches, so far as they related to the putting up of artificial dentures, could be acquired in the laboratory of the dental practitioner, and under his immediate supervision, but success in operating upon the teeth *in situ* could not be attained in this way. It was only in rare cases, that a dental surgeon could prevail upon a patient to allow the presence of a third person, in the shape of a young student, who made it his business to be very inquisitive as to what was going on, and who was continually asking information relative not only to the operation then being performed, but to all the operations which ever had, or ought to have been performed in the patient's mouth. If the student made everything he did not understand the subject of inquiry, he was apt to be considered as impertinent, and if he did not do so, (and on the spot too,) he most certainly lost opportunities of gaining informatian, which to him would have been most valuable, and like opportunities might not again occur. But taking it for granted, that the student had made the best of his facilities for gaining information, he was still deficient in the most important particular. No dentist who can really well perform that exceedingly difficult and valuable operation, known as filling a tooth, will pretend to say that it can be taught properly by mere appeals to the sight and mind. It can only be acquired by actual manipulation on the part of the learner, and unless he is constantly watched and guided by some person of skill, experience and judgment, he will be likely to do much injury to his patient, and to make but slight progress himself. The duties devolving upon the dentist in his laboratory, were such as enabled him to impart his knowledge of the mechanical department, while engaged in the performance of these duties, and that too, in the most practical manner, for the student could work with and be guided by him. Not so with operative dentistry, in the office there could be but one patient, and for that patient, but one operator—for how many were willing to pay for dental operations, and then be made the subject for clinical lectures ? or what dentist could afford to spend his time, in superintending the operations of his student upon a patient who received the benefit gratis. Another great difficulty in the way, was the fact, that the reputation of the teacher, as a general rule, depended more upon his success in practice, than his ability to impart knowledge. As successful practice was necessarily an extensive one, hence a greater number of students, and a proportionably *diminished* time to devote to their interests.

An experiment had been made of teaching dentistry by means of lectures in medical colleges ; but it became obvious to those who attempted it, that it was ineffectual, so far as the main object was concerned, and was attended with one disadvantage which would of itself more than counterbalance the good to be derived. The proportion of those attending medical colleges for the benefit of dental lectures, was exceedingly small ; and those who attended for the purpose of perfecting themselves in the various branches of medicine, took but slight interest in the dental course. Dental students, in like manner, gave their attention to their own branch at the expense of the medical lectures. It hence became obvious, that though good physicians might be sent forth from the college, that good dentists could not; while those who studied the dental specialty would not be skillful physicians. How then could the *public* distinguish between the parties, *both coming before it with the same title to practice in either profession?* What was to hinder the physician without a practice from filling his pockets with shining teeth, or the dentist from trifling with diseases of which he knew barely the name. There were some few members of the profession then as now, whose interest, it may be, caused them still to advocate the plan, ineffectual as it was; and they, too, asked, what was to hinder the physician or any one else from commencing the practice of dental surgery without due preparation? It is true, there was no law to prevent it; but those dentists who had the welfare of the public and profession at heart, knew that some guarantee should be given—some distinction should be made, so that patients might know in whom to confide. This course, alone, could secure to the profession the position to which it was entitled, and nothing less than this could purge it of many of its unworthy members. A distinction of any kind that could be recognised by the public, would, if bestowed only upon the worthy, force those who were not entitled to receive it either to qualify themselves or give up the practice of a profession they disgraced.

In 1840, the Baltimore College of Dental Surgery held its first session. The charter of this institution empowered its officers to bestow the degree of Doctor of Dental Surgery upon its graduates. About the same time, the American Society of Dental Surgeons was established "for the mutual improvement of its members and the elevation of the profession." It was necessary that the applicant for membership should have practiced a certain length of time and should pass an examination before a committee appointed for that purpose, ere he could be considered a member. The formation of this society was soon followed by others in different parts of the country; all of them, however, having objects somewhat similar and tending more towards the benefit of the practitioner than the student.

The method of instruction pursued in the Baltimore College of Dental Surgery, has now been in successful operation something over ten years; it was not adopted without due consideration on the part of its faculty. A sufficient proof of its superiority over the plan proposed of adding a course of dental lectures to the regular lectures of the medical colleges, is given in the fact of Dr. H. H. Hayden's acceptance of one of the chairs of the dental college—*after having tried the former plan and found it wanting*. The faculty of the Baltimore College of Dental Surgery consists of

Professor of Principles and Practice of Dental Surgery.
" Operative and Mechanical Dentistry.
" Special Pathology and Therapeutics.
" Anatomy and Physiology.
Demonstrator of Mechanical Dentistry and Lecturer on Chemistry.

*Three* of the above offices are filled by practicing dentists, and two (special pathology and anatomy) by physicians. Lectures in the four departments, named above, are delivered each afternoon. The mornings are especially devoted to the *practice* of the various branches. The mechanical department being superintended by the demonstrator; and the operations in the infirmary by the professor of operative and mechanical dentistry. The infirmary attached to the institution furnishes every variety of case to be treated by the dental surgeon. Upon the patients thus procured, the students operate, being personally superintended and guided by the officer in attendance. By this means, students gain confidence, skill and dexterity in manipulation, as well as actual experience. These were the points of dental education that were not reached by any of the previous methods, and they are certainly of paramount importance. During the session of 1850,-1, upwards of seventeen hundred cases were treated by the students of this college. This fact is, of itself, so impressive, that we shall make no further remark upon it. At the present time, the number of graduates from this institution is about one hundred—of these, nineteen had previously earned the degree of Doctor of Medicine. The examination of candidates, for the honors of the institution, takes place in the presence of a committee chosen for that purpose, from the most prominent members of the profession. After the candidates have been examined in the more important points by the faculty, they are handed over to the tender mercies of the committee, who, by the way, have opportunities of examining into the relative merits and demerits of the operations performed and the mechanical work put up—each student being obliged to produce specimens of his skill in both departments for such examination.

Now, we consider this method of education, somewhat thorough. If any think it can be improved, let them speak out. Improvement is the order of the day. But before improvements are *attempted*, let us know in what *particular* the present system is imperfect. It certainly has not failed, as some have insinuated ; on the contrary, its prospects have brightened every year, and those prospects have been realized. The number of students has been slowly, but constantly increasing—each class has evinced an improvement on the part of its members over the class of previous years ; a result of the increased experience of the faculty in teaching and the favorable reputation which the infirmary has obtained in the estimation of the public, thus obtaining for the students a constantly increasing number of patients.

A college of dental surgery has been in operation some six years in Cincinnati. It has labored under greater difficulties, and hitherto has not met with success equal to that of its elder sister, still it has not failed. Within the past six months, the college buildings, apparatus, &c. have been purchased by a number of dentists, residing principally, we believe, in the west ; and we may now consider the institution as standing upon a firmer foundation than ever ; as a greater number of dentists are directly interested in its success than was previously the case. Of the graduates of the Ohio Dental College, over one half had practiced from four to twenty years, at the time they entered the institution. The same can probably be said of the college in Baltimore ; a most convincing proof that *something* is taught in these institutions, that cannot be taught in the office of a dentist, and cannot readily be attained in the ordinary course of a dental practice of many years.

Dr. C. A. Harris, tells us, in a late number of the Journal, that there is a general tendency evinced by dental students to devote extra time to the practical departments at the expense of the regular lectures, and that it has required the stringent laws of the institution, and effort on the part of the faculty, to counteract this tendency. Dr. James Taylor, in a letter now before me, says, "I have found a greater disposition (on the part of the students) to obtain merely mechanical knowledge than I had hoped to see." Again, speaking of the classes, "the first session we had over *twenty students*, *since then*, classes have ranged from *eight* to *fifteen*." "*The first session students were not obliged to take all the tickets*, *subsequently* we have allowed *none but full course students*, and hence those seeking only *mechanical knowledge were shut out*." "Our object has been more to advance the interests of the profession than to obtain large classes." We certainly think that Dr. T. and his worthy co-laborers, deserve the thanks of the profession. They have worked hard and long, and we hope they will soon be reaping the reward of their labors. One thing is certain, the experiment has not yet proved a failure ; on the contrary, it has been so far successful as to induce others to enter the field; arrangements are now making for the establishment of two new dental colleges, and we heartily agree with a gentleman standing high among dentists, and from whose letter we quote, "there is no possible reason why dental colleges should not be *well sustained*, and nothing but a jealous, selfish feeling on the part of some members of the profession can prevent it. This, time must overcome ; no man properly regarding the true interests of his profession, can long entertain these feelings." "If dentists believe that these institutions are not properly conducted, they should at once set to work to rectify the evil." To this last quotation we would add our opinion that *dentists generally cannot but feel satisfied* with the manner in which dental colleges have been and are being conducted.

